# Synthesis of silicalite-poly(furfuryl alcohol) composite membranes for oxygen enrichment from air

**DOI:** 10.1186/1556-276X-6-637

**Published:** 2011-12-30

**Authors:** Li He, Dan Li, Kun Wang, Akkihebbal K Suresh, Jayesh Bellare, Tam Sridhar, Huanting Wang

**Affiliations:** 1 Department of Chemical Engineering, Monash University, Clayton, Victoria, 3800, Australia; 2 Department of Chemical Engineering, Indian Institute of Technology Bombay, Bombay, Maharashtra,400076, India

**Keywords:** poly(furfuryl alcohol), silicalite, composite membrane, air separation

## Abstract

Silicalite-poly(furfuryl alcohol) [PFA] composite membranes were prepared by solution casting of silicalite-furfuryl alcohol [FA] suspension on a porous polysulfone substrate and subsequent *in situ *polymerization of FA. X-ray diffraction, nitrogen sorption, thermogravimetric analysis, scanning electron microscopy, and energy-dispersive X-ray spectroscopy were used to characterize silicalite nanocrystals and silicalite-PFA composite membranes. The silicalite-PFA composite membrane with 20 wt.% silicalite loading exhibits good oxygen/nitrogen selectivity (4.15) and high oxygen permeability (1,132.6 Barrers) at 50°C. Silicalite-PFA composite membranes are promising for the production of oxygen-enriched air for various applications.

## Introduction

Oxygen-enriched air can be widely used in chemical industries, fermentation, biological digestion processes, and medical purposes [1-3]. For example, combustion with oxygen-enriched air can substantially reduce fuel consumption and improve energy efficiency, thereby lowering CO_2 _emission [2].

The cryogenic fractionation technology is commonly used to produce oxygen-enriched air with an oxygen purity of 99 vol.%. Pressure swing adsorption can yield 95 to 97 vol.% oxygen-enriched air [4]. The membrane technology has also been researched for oxygen separation from air. Over the past decades, polymeric gas separation membranes have attracted much attention, becoming one of the fastest growing branches of membrane technology. This is because polymeric membranes tend to be relatively inexpensive and can be easily fabricated into hollow fibers or spiral-wound modules [5,6]. Some polymeric membranes such as silicone rubber, polyphenylene oxide, and cellulose triacetate have already been studied for oxygen enrichment [2,3,7]. However, because the molecular dimensions of O_2 _(3.46 Å) and N_2 _(3.64 Å) are close, producing pure oxygen is rather difficult as some nitrogen always permeates through the membrane [5]. The separation properties of existing polymeric membranes are still restricted by the trade-off trend between gas permeability and selectivity which was suggested by Robson [8]. Additional limitation of the polymeric membrane is that at elevated temperatures, the performance of the membrane will lose because of the segmental flexibility [9]. Therefore, the separation membranes with high O_2_/N_2 _selectivity and high flux are required to be competitive with other technologies.

Inorganic molecular sieves (such as zeolites) exhibit good chemical and thermal stabilities and high gas flux and selectivity, but the fabrication of defect-free molecular sieve membranes remains a challenge. In recent decades, there has been significant interest in the development of synthesis methods of pinhole-free, mechanically stable, and inorganic-organic hybrid membranes to combine the advantages of both inorganic and organic membranes. Such kind of membrane is known as mixed matrix (or composite) membranes. Desirable composite membranes consist of well-dispersed particles without interfacial incompatibility and defects between the inorganic material and the polymer. Therefore, careful selection of a pair of polymer and inorganic material is very important. Polysulfones, polyarylates, polycarbonates, poly(arylethers), poly(arylketones), and polyimides are frequently used in industrial polymeric membrane gas separations. The commonly used inorganic materials include carbon molecular sieves, zeolites, mesoporous materials, activated carbons, carbon nanotubes, and metal-organic framework [10].

In the present work, we attempt to develop zeolite-polymer composite membranes for oxygen production from air. In particular, poly(furfuryl alcohol) [PFA] is chosen as the polymer matrix, and silicalite, as the molecular sieve additive. In the work previously conducted by one of the authors (Wang et al.), poly(furfuryl alcohol) was used to prepare a zeolite 4A polyfurfuryl alcohol nanocomposite membrane by vapor deposition polymerization of furfuryl alcohol [FA] [11,12]. This composite membrane showed an O_2_/N_2 _selectivity as high as 8.2 and an oxygen permeance of 1.5 × 10^-9 ^mol·m^-2^·s^-1^·Pa^-1^. To improve oxygen flux, silicalite is used in the present study because it has a larger pore size than the zeolite 4A. Silicalite is a pure silica MFI-type zeolite, which is composed of a uniform molecular-sized pore system with straight channels in the b-direction (5.4 Å × 5.6 Å) and sinusoidal channels in the a-direction (5.1Å × 5.5 Å) [13,14].

## Experimental details

### Materials

One molar tetrapropylammonium hydroxide [TPAOH] aqueous solution and tetraethyl orthosilicate [TEOS] (99%), acrylamide [AM], *N,N'*-methylenebisacrylamide [MBAM], and FA (98%) were purchased from Sigma-Aldrich Corporation (Sydney, Victoria, Australia). A polysulfone ultrafiltration membrane (MWCO 30,000) was purchased from Sterlitech (GE Osmonics, Minnetonka, MN, USA) and used as the support.

### Preparation of silicalite nanocrystals

Silicalite nanocrystals were synthesized according to the previously published procedures [15]. In a typical synthesis, silicalite nanocrystals were synthesized by hydrothermal synthesis from a clear solution with a molar composition of 1 TPAOH:4.8 SiO_2_:44 H_2_O. The synthesis solution was prepared in a 250-mL polypropylene bottle. First, 20 g of 1 M TPAOH solution was added dropwise into 20 g of TEOS under vigorous stirring. Strong magnetic stirring was maintained for 3 h. The solution was then heated in an oven at 80°C for 5 to 6 days for crystallization, resulting in a milky silicalite suspension. The solid product contained in the colloidal suspension was recovered by a repeated cycle of centrifugation with deionized water and ultrasonic redispersion in water until pH < 8. An organic polymer network was prepared from water soluble organic monomers, AM and MBAM, and the initiator (NH_4_)_2_S_2_O_8 _as a temporary barrier during calcination and carbonization to obtain highly redispersible template-free silicalite nanocrystals. Typically, 1.0 g of AM, 0.1 mg MBAM, and 25 mg of (NH_4_)_2_S_2_O_8 _were added under stirring into 10 g of silicalite colloidal suspension with 5 wt.% solid loading. After the monomers were dissolved, the mixture was ultrasonicated to ensure complete dispersion of silicalite nanocrystals for half an hour. The aqueous solution was then heated at 50°C for 30 min to be polymerized into an elastic hydrogel. This silicalite hydrogel polymer composite was dried at 80°C overnight. After drying, it was carbonized under nitrogen at 550°C for 2 h (heating rate, 2°C min^-1^) and then calcined at 550°C for 3 h under air.

### Preparation of silicalite/PFA composite membranes

Both plain PFA and silicalite-PFA composite membranes were hand-cast on commercial polysulfone ultrafiltration membranes. [16] A 25 mm × 70 mm polysulfone ultrafiltration membrane was fixed on the top of a microscope glass slide using a tape to prevent the membrane from rolling up and solution penetration through the edges. Then, an aqueous solution prepared by mixing 10 g of FA and 0.04 g of sulfuric acid with 10 g of ethanol was cast on the polysulfone membrane substrate for 5 min at room temperature. The coated support was then heated at 80°C overnight for FA polymerization. Silicalite-PFA nanocomposite membranes were made using the same procedures, except that a given amount of template-free silicalite nanocrystals was dispersed in the FA ethanol solution which was ultrasonicated for 30 min at room temperature. The resulting silicalite-FA ethanol suspension was immediately mixed with sulfuric acid under magnetic stirring for 2 min and then coated on the polysulfone membrane substrate for 5 min at room temperature. The coated support was heated at 80°C overnight. The resultant composite membranes are referred to as 1-Sil-PFA, 10-Sil-PFA, 20-Sil-PFA, and 30-Sil-PFA, corresponding to silicalite loadings of 1% (*w*/*w*), 10% (*w*/*w*), 20% (*w*/*w*), and 30% (*w*/*w*) in PFA solution, respectively.

### Characterization

X-ray diffraction [XRD] patterns were recorded on a Philips PW1140/90 diffractometer (PANalytical B.V., Almelo, The Netherlands) with Cu Kα radiation (25 mA and 40 kV) at a scan rate of 2°/min with a step size of 0.02°. Nitrogen adsorption-desorption experiment was performed at 77 K and at room temperature with a Micrometritics ASAP 2020MC analyzer (Micromeritics Instrument Co., Norcross, GA, USA). To evaluate the thermal stability of the PFA, thermogravimetric analysis [TGA] was conducted using a thermogravimetric analyzer (PerkinElmer, Waltham, MA, USA) in the temperature range of 20°C to 700°C under nitrogen gas and a heating rate of 5°C/min. All scanning electron microscopy [SEM] images were taken with a FEG-7001F microscope (JEOL, Ltd., Akishima, Tokyo, Japan) operated at an accelerating voltage of 15 kV. Elemental analysis of samples was determined by energy dispersive X-ray spectroscopy [EDXS] on the FEG-7001F microscope.

### Gas separation

The PFA composite membrane samples were dried at 80°C overnight before the gas permeation test. The single gas permeation of membranes was measured using the pressure rise method. The feed gas was supplied at room temperature and atmospheric pressure. The permeate rate was determined by isolating the permeate volume from the vacuum supply and subsequently monitoring the pressure change in the permeate side. The effective membrane area was 0.95 cm^2^. The pressure rise was recorded by a MKS 628D Baratron transducer (MKS Instruments Inc., Wilmington, MA, USA). Membrane permeability, *P_i _*(Barrer; 1 Barrer = 10^-10 ^cm^3 ^(STP)·cm·cm^-2^·s^-1^·cmHg^-1^), was defined as [1,17-19]

$$P_{i}=\frac{dN_{i}}{\Delta p_{i}A},$$

where *N_i _*(mol·s^-1^) is the permeate flow rate of component gas *i*, Δ*p_i _*(in Pascals) is the transmembrane pressure difference of *i*, and *A *(in square centimeters) is the membrane area.

The ideal selectivity *α_ij _*was calculated from the relation between the permeance of pure *i *and *j *gases [1,17]:

$$\alpha_{ij}=\;\frac{P_{i}}{P_{j}}.$$

The apparent activation energy *E*_p _(in kiloJoule per mole) was analyzed according to the Arrhenius equation [20-23]:

$$P=P_{\text{o}}\text{exp }\left[\frac{-E_{\text{p}}}{RT}\right],$$

where *P *is the permeability (in Barrers), *P*_o _is the pre-experiential factor, *T *is the absolute temperature (in Kelvin), and *R *is the gas constant (8.3143 J·mol^-1^·k^-1^).

The volume fraction of oxygen *X*_02 _in the product gas from air is shown in the following equation [24]:

$$X_{\text{O2}}=\frac{1}{2\left\{\frac{\left(\alpha -1\right)\left(0.21+\varphi \right)+1}{\left(\alpha -1\right)\varphi }-\sqrt{{\left(\frac{\left(\alpha -1\right)\left(0.21+\varphi \right)+1}{\left(\alpha -1\right)\varphi }\right)}^{2}-\frac{\left(4\right)\left(0.21\right)r}{\left(\alpha -1\right)\varphi }}\right\}},$$

where *α *is the selectivity of O_2 _to N_2_, *φ *is the ratio of product to feed gas pressures, and 0.21 is the fraction of oxygen in the feed air.

## Results and discussion

### Silicalite nanocrystals and silicalite-PFA membranes

**Figure 1:**
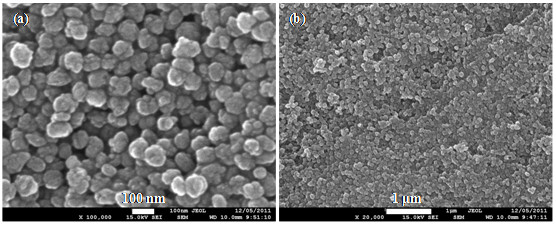
**SEM image of silicalite nanocrystals**.

N_2 _adsorption measurement shows that silicalite nanocrystals have a BET surface area of 404 m^2^/g and a t-plot microspore volume of 0.12 cm^3^/g, which are close to the reported values for crystalline nanosilicalite [15,25]. The SEM images of silicalite nanocrystals shown in Figure 1 reveal uniform spherical nanoparticles and a narrow particle size distribution ranging from 60 nm to 100 nm.

**Figure 2:**
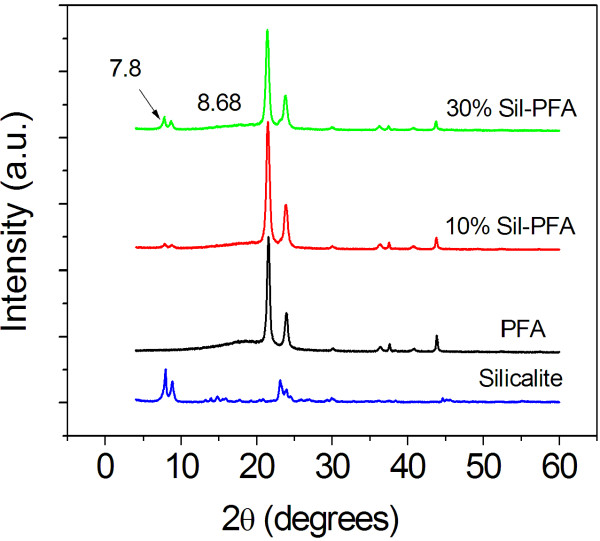
**XRD patterns of silicalite, PFA membrane, and Sil-PFA composite membranes with different silicalite loadings**.

Figure 2 shows XRD patterns of the silicalite, PFA composite membrane, and Sil-PFA composite membrane with different silicalite loadings (10 wt.% and 20 wt.%). The diffraction peaks at 2*θ *= 21.58, 24.13, 30.10, 36.38, 40.92, and 44.18 arise from the substrate that consists of a polysulfone membrane supported on a polyethylene non-woven polyethylene fabric. These peaks are mainly from the polyethylene fabric [26]. For silicalite nanocrystals, there are two peaks at 2*θ *= 7.8 and 2*θ *= 8.68. When the loading of silicalite nanocrystals in Sil-PFA composite membranes increases, the intensities of these two peaks increase. This confirms the presence of silicalite nanoparticles in the PFA polymer.

**Figure 3:**
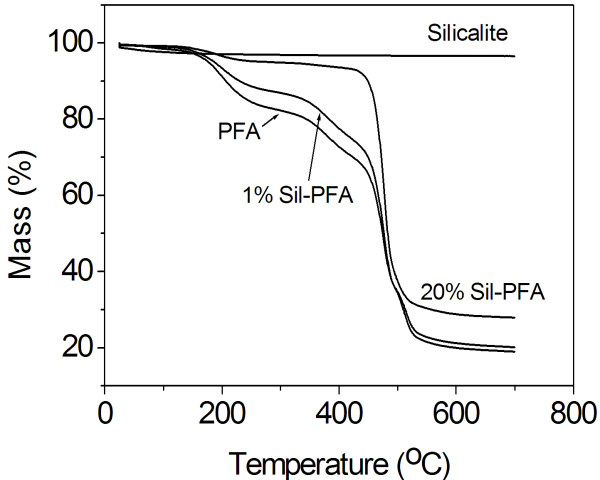
**TGA curves of silicalite nanocrystals, polysulfone substrate and PFA, and silicalite-PFA composite membranes**.

Thermogravimetric analysis of the silicalite nanocrystals, PFA, and silicalite-PFA composite membrane (Figure 3) shows that the silicalite crystals remain in the testing temperature under N_2_, except a slight mass loss (*ca*. 2.9%) at 100°C to 200°C which is attributed to water desorption. However, under flowing nitrogen, the PFA composite membrane loses *ca*. 8.9% of its mass in the temperature range from 25°C to 200°C, corresponding to the loss of absorbed water. In the temperature range of 200°C to 450°C, there is a 25.7% mass loss. From 450°C to 700°C, a further 46.4% mass loss is observed due to the decomposition of the PFA membrane. The mass losses of Sil-PFA composite membranes are much slower than those of the PFA composite membrane. The total final mass losses are 81.0%, 79.8%, and 72.1% for pure PFA, 1% Sil-PFA, and 20% Sil-PFA composite membranes, respectively. The mass losses of the supported PFA and silicalite-PFA composite membranes are much smaller than those of the pure PFA. This result indicates that the Sil-PFA composite membrane with high silicalite loading exhibits higher thermal stability.

**Figure 4:**
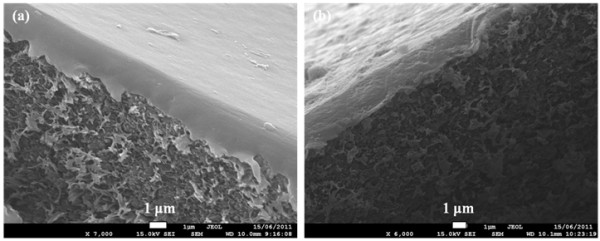
**SEM images of the cross-section view of the membranes**. (**a**) PFA composite membrane and (**b**) 10% Sil-PFA composite membrane.

**Figure 5:**
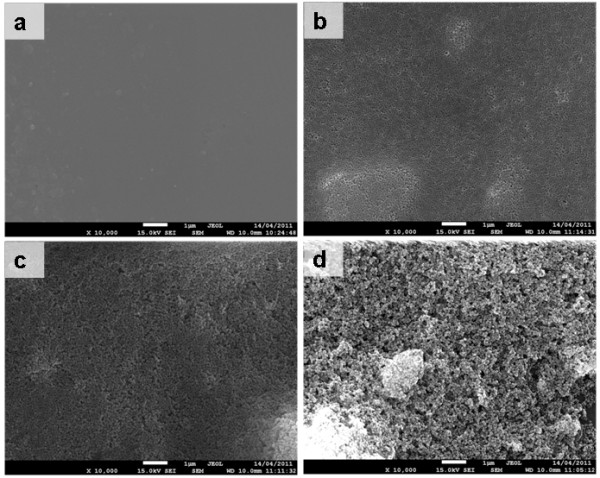
**SEM images**. (**a**) PFA, (**b**) 10% Sil-PFA, (**c**) 20% Sil-PFA, and (**d**) 30% Sil-PFA composite membranes. Scale bar = 1 μm.

Figure 4 shows SEM images of the cross-sections of the pure PFA membrane and 10% Sil-PFA composite membrane. The thicknesses of the active PFA layer and silicalite-PFA are around 1.5 to 2.0 μm. The cross-section morphology reveals the strong adhesion among PFA and the silicalite nanoparticles and the substrate, and that the silicalite particles are well dispersed in the PFA. Figure 5 shows SEM images of the top surfaces of the pure PFA composite membrane and 10% Sil-PFA, 20% Sil-PFA and 30% Sil-PFA composite membranes. The PFA membrane exhibits a smooth surface (Figure 5a), which is similar to that reported previously [27]. Uniform dispersion of silicalite nanocrystals throughout the Sil-PFA composite membrane surfaces was clearly observed (Figure 5b, c). However, as the concentration of silicalite increases to 30% (*w*/*w*), the agglomeration of silicalite nanoparticles becomes evident (Figure 5d).

**Figure 6:**
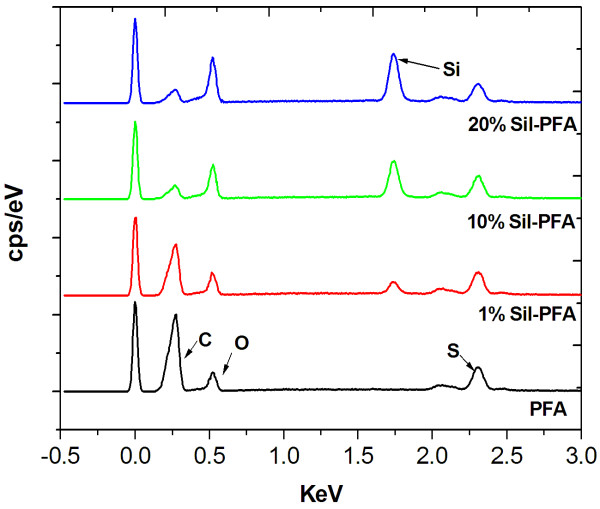
**EDX spectra of PFA, 1 wt.% Sil-PFA, 10 wt.% Sil-PFA, and 20 wt.% Sil-PFA composite membranes**.

The presence of silicalite nanocrystals was also confirmed by EDX. In Figure 6, the carbon, oxygen, silicon, and sulfur peaks in EDX spectra arise from polysulfone-supported PFA composite membranes, and there is no silicon peak in the plain PFA composite membranes. Then, the Si peak appears at approximately 1.74 keV in 1% Sil-PFA composite membranes. As the loading of silicalite increases, the intensity of silicon peak increases. This clearly indicates the presence of silicalite nanocrystals in the Sil-PFA composite membranes.

### Gas separation properties

Table 1 summarizes the permeability values of single N_2 _and O_2 _gases and the O_2_/N_2 _ideal selectivity for plain PFA, 1% Sil-PFA, 10% Sil-PFA, 20% Sil-PFA, and 30% Sil-PFA composite membranes. Single gas permeation experiments showed that the permeability of both gases increased largely with the increasing silicalite loading. For example, O_2 _permeability increases from 3.3 Barrers for the plain PFA membrane to 966.4 Barrers for the 30% Sil-PFA composite membrane. The O_2_/N_2 _ideal selectivity increases from 1.25 for the plain PFA membrane to 3.52 for the 20% Sil-PFA and then drops to 1.06 for the 30% Sil-PFA. The selectivity of the 20% Sil-PFA composite membrane is almost three times greater than that of the plain PFA membrane. Compared to the O_2_/N_2 _upper bound data in the relationship of permeability and selectivity in the reference [8], the O_2_/N_2 _separation characteristics of the 20% (*w*/*w*) Sil-PFA composite membrane is on the prior upper bound. In the literature, the O_2_/N_2 _separation PIM-1 membrane was 4.0 at an O_2 _permeability of 370 Barrers [28]. Poly[1-phenyl-2-*p*-(trimethylsilyl)phenylacetylene] membrane had an O_2 _permeability of 1,550 Barrers and an O_2_/N_2 _separation of 2.98 [29]. In our case, high gas permeability may be contributed from diffusion of gas through large pores (5.5 Å) of the silicalite. The low O_2_/N_2 _selectivity observed in the composite membrane with 30% silicalite loading should be due to silicalite-PFA interfacial defects [30]. Likewise, agglomeration may also occur during the membrane fabrication. This agglomeration leads to small pinholes which are not filled up with PFA polymer, forming nonselective defects in the composite layer [30].

**Table 1:** Gas permeation results of PFA and composite membrane

Sample	Silicalite loading (%)	Permeability (Barrers^a^)		O_2_/N_2 _ideal selectivity
		N_2_	O_2_	
PFA	0	2.67	3.33	1.25
1% Sil-PFA	1	20.7	27.7	1.34
10% Sil-PFA	10	64.0	185.3	2.90
20% Sil-PFA	20	233.3	821.2	3.52
30% Sil-PFA	30	913.12	966.4	1.06

^a^1 Barrer = 1 × 10^-10 ^cm^3^(STP) cm/cm^2^·s·cm·Hg

Table 2 summarizes the permeability values of single N_2 _and O_2 _gases and O_2_/N_2 _ideal selectivity for the 20 wt.% silicalite loading Sil-PFA composite membrane at different testing temperatures ranging from 20°C to 150°C. Single gas permeation experiments showed that as the temperature was increased from 20°C to 50°C, both permeabilities and selectivities increased; whereas the permeability of all gases still increased, but their selectivity decreased as the temperature further increased from 50°C to 150°C. For example, at a testing temperature of 20°C, the membrane had an oxygen permeability of 233.3 Barrers and an O_2_/N_2 _selectivity of 3.52. As the temperature increased to 50°C, oxygen permeability increased to 233.3 Barrers, and O_2_/N_2 _selectivity also increased to 4.15. At 150°C, the oxygen and nitrogen permeabilities were about 2.6 and 3.8 times higher than those at 20°C, respectively, but the O_2_/N_2 _selectivity was about 1.5 times lower. Furthermore, the nitrogen permeability at 150°C was also much higher than that at 20°C.

**Table 2:** Gas permeation results of the 20% Sil-PFA composite membrane at different testing temperatures

Temperature (°C)	Permeability (Barrers)		O2/N2 ideal selectivity
	N2	O2	
20	233.3	821.2	3.52
50	276.3	1132.6	4.15
100	538.7	1926.9	3.58
150	903.5	2135.2	2.36

The values of the apparent activation energy for nitrogen and oxygen permeation through PFA and 20% Sil-PFA membranes are presented in Table 3. It is apparent that N_2 _molecules require more energy to penetrate through the membranes than O_2_. In particular, the activation energy for N_2 _permeation through the composite membrane is only slightly smaller than that for the plain PFA membrane; however, the activation energy for O_2 _is much smaller for the composite membrane than that for the PFA membrane. This suggests that incorporating the silicalite particles into the PFA matrix can largely reduce the energy barrier for O_2 _permeation through the membrane, therefore improving O_2 _flux, and O_2_/N_2 _selectivity.

**Table 3:** Apparent activation energy for permeation of N_2 _and O_2 _gases

Sample	Apparent activation energy (kJ/mol)	
	N2	O2
PFA	12.03	11.65
20% Sil-PFA	11.90	7.99

**Figure 7:**
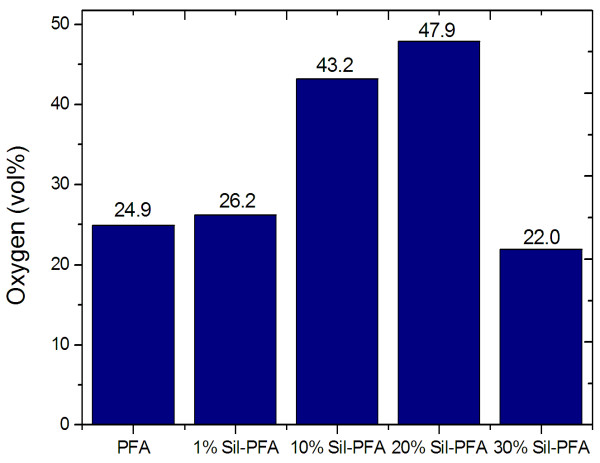
**The volume percentage of oxygen in the product gas from air**.

Figure 7 shows the effect of the silicalite loading on the volume percentage of oxygen in the product gas from air. At room temperature, O_2_-enriched air containing 47.9 vol.% O_2 _was obtained when the 20% silicalite-PFA composite membrane was used.

## Conclusions

Silicalite-PFA composite membranes were prepared for enrichment of oxygen from air. SEM results showed that silicalite nanoparticles were well dispersed in the PFA matrix. The gas permeation experiments indicated that O_2 _and N_2 _permeabilities and O_2_/N_2 _selectivity could be improved by incorporating silicalite nanoparticles into PFA. In particular, the Sil-PFA composite membrane with 20% silicalite loading had the highest O_2_/N_2 _selectivity and excellent O_2 _permeability, and an oxygen concentration of 47.9 vol.% was achieved in the single-pass air separation experiment at room temperature.

## Competing interests

The authors declare that they have no competing interests.

## Authors' contributions

LH carried out most of the experimental work including the membrane preparation, characterization, and gas permeation testing and drafted the manuscript. DL was involved in designing the gas permeation experiments, and KW helped with the electron microscopy experiments. HW revised the manuscript. AKS, JB, and TS were involved in the discussions of experimental results. All authors read and approved the manuscript.

## Acknowledgments

This work was supported by the Department of Innovation Industry, Science and Research of Australian Government through the Indo-Australian Science and Technology Fund and the Australian Research Council. The authors gratefully acknowledge the support and use of facilities in the Monash Centre for Electron Microscopy. Huanting Wang thanks the Australian Research Council for a Future Fellowship.
